# Comparison of efficacy of corticosteroid injection versus extracorporeal shock wave therapy on inferior trigger points in the quadratus lumborum muscle: a randomized clinical trial

**DOI:** 10.1186/s12891-020-03714-3

**Published:** 2020-10-19

**Authors:** Bina Eftekharsadat, Negar Fasaie, Dina Golalizadeh, Arash Babaei-Ghazani, Fatemeh Jahanjou, Yashar Eslampoor, Neda Dolatkhah

**Affiliations:** 1 Physical Medicine and Rehabilitation Research Center, Aging Research Institute, Tabriz University of Medical Sciences, Tabriz, Iran; 2 Faculty of Medicine, Tabriz University of Medical Sciences, Tabriz, Iran; 3 Neuromusculoskeletal Research Center, Department of physical medicine and rehabilitation, Iran University of Medical Sciences, Tehran, Iran; 4 Tabriz University of Medical Sciences, Tabriz, Iran; 5 Palliative Care Medicine Department, Faculty of Medicine, Tabriz University of Medical Sciences, Tabriz, Iran

**Keywords:** Extracorporeal shockwave therapy, Corticosteroid, Trigger point, Quadratus Lumborum

## Abstract

**Background:**

In this study, we aimed to compare the efficacy of corticosteroid trigger point injection (TPI) versus extracorporeal shock wave therapy (ESWT) on inferior trigger points in the quadratus lumborum (QL) muscle.

**Methods:**

In this single-blind randomized clinical trial, 54 low back pain patients with myofascial trigger points on QL muscle were investigated. Participants were randomly allocated into two groups with A and B pockets. Patients in group A underwent radial ESWT and received 5 treatment sessions (1 per week) and actually were not followed-up. However, patients in group B received corticosteroid TPI and received one session of corticosteroid treatment and followed-up for 4 weeks after injection. Oswestry Disability Index (ODI), visual analogue scale (VAS), pain pressure threshold (PPT) and short form (36) health survey (SF-36) were measured in both groups before, two weeks after and four weeks after intervention.

**Results:**

The between group comparison indicated that corticosteroid TPI leaded to significant higher improvements of ODI (*P*-value< 0.01), VAS (*P* value< 0.001), and PPT (P-value = 0.001) scores compared to the ESWT group at two-week follow-up time-point. ESWT group recorded significant higher improvement of ODI (P-value< 0.01) and SF-36 (P-value< 0.001) compared to the corticosteroid TPI at 4th week post treatment evaluation. At four-week follow-up time-point, the patients in the ESWT group were 1.46 times more likely to achieve 30% reduction in VAS, 2.67 times more likely to achieve 30% reduction in ODI, and 2.30 times more likely to achieve 20% improvement in SF-36 compared to the participants in corticosteroid TPI group. These results refer to large effect size for all study outcomes in ESWT group (d = 4.72, d = 1.58, d = 5.48, and d = 7.47 for ODI, PPT, SF-36, and VAS, respectively).

**Conclusion:**

Corticosteroid TPI was more effective compared to ESWT in short-term controlling of pain and disability caused by myofascial pain syndrome of QL muscle. However, after 4 weeks treatment, ESWT further improved the quality of life and disability and was related with more probability of achievement the minimal clinically important difference concerning pain, disability and quality of life and large effect size for all study outcomes in treated patients compared to corticosteroid TPI.

**Trial registration:**

www.irct.ir, IRCT20100827004641N14, retrospectively registered 2019-01-19.

## Background

Low back pain (LBP) is a common health issue in all over the world, especially in the industrialized countries. Approximately 80% of people experience LBP at least once in their life [1]. Although LBP initiates with mild limited pain, it may cause complications in case of incidents, in a way that nearly 15% of the patients with LBP may be presentably disabled. Performing physical activity and exercise therapy in LBP is paid much attention in the last few decades [2, 3]. Too much using and straining of the quadratus lumborum (QL) muscle is one of the main reasons of chronic LBP. This muscle is located lateral to spine in the lumbar vertebrae region, which is attached to the inferior edge of 12th rib and transverse process of the first 4 lumbar vertebrae on the one side, and medial surface of iliac crest on the other side. Myofascial pain syndrome (MPS) of QL is among treatment resistant etiologies of LBP [4].

Trigger point is a point with high irritability inside a taut band of skeletal muscle, which can cause a certain pattern of radiating pain and tenderness when exposed to pressure or stretching [5–7]. Trigger points may be organized in any muscle; however, trigger points are more common in muscles involved in the body balance [8]. Myofascial trigger point has a multifactorial etiology, which most importantly includes psychological factors, inappropriate biomechanics, and muscle overuse [5, 8]. There are several methods for resolving of myofascial trigger points, including non-surgical interventions such as NSAIDs, dry needling, avoiding severe activities, and using physical modalities such as trigger point injection (TPI) and extracorporeal shock wave therapy (ESWT) [9]. Local analgesic, saline, corticosteroid, botulinum toxin, and dry needling methods are diverse injection methods that can be applied to deactivate the trigger points [10]. Local corticosteroid injection, inhibits the phagocytosis and synthesis and release of inflammatory chemical mediators and enzymes, and in this way can exert anti-inflammatory effects while protecting the patient from systemic side effects [11].

Since mid-1990s, shockwave is used in the treatment of some musculoskeletal disorders such as lateral epicondylitis, shoulder calcification, and plantar fasciitis. In ESWT, waves are formed with electromagnetic, piezoelectric, and electrohydraulic methods. ESWT has been considered as an alternative therapeutic approach for MPS over the last 25–30 years, especially in the subjects with symptoms resistant to conventional treatments [12]. Hye Min Ji et al. [13] assessed the efficacy of ESWT in the upper trapezius MPS and established that, ESWT significantly decreased the pain intensity in treated subjects.

So far, evidences on ESWT’s efficiency in QL muscle are limited, and on the other hand the effect of ESWT has not been compared with corticosteroid injection in the lower back trigger points in nonspecific LBP. Hong et al. [14] has recently compared the effectiveness of ESWT and TPI for the treatment of MPS in the QL; however, this study was a retrospective study, not a randomized clinical study, and the participants’ interests to the treatment technique may lead to confounded and biased findings. Thus, we decided to the prospectively compare the effectiveness of corticosteroid TPI and ESWT in the inferior trigger points of the QL muscle.

## Methods

### Study design and setting

This study was an assessor-blinded, parallel-group, randomized controlled trial with a 1:1 allocation ratio, which was conducted at the Shohada Educational Hospital in 2019, Tabriz, Iran. The study was approved by the ethics committee of Tabriz University of Medical Sciences. The research was conducted in terms of the Helsinki Declaration, and informed written consent was obtained from all participants. The CONSORT guidelines were conformed and the CONSORT diagram was applied to demonstrate the flow of participants at each stage of the study.

### Study sample

The inclusion criteria were the presence of LBP for at least three months [15, 16], at least one local tenderness or active trigger point in the inferior anatomic region of the QL muscle, which can be distinguished by pain, referred pain, and local twitch response by gentle manual compressing [17–19] and a palpable nodule along with a taut band in the selected muscles based on the anatomical position (MPS established in terms of the criteria specified by Travel and Simon) [20], normal neurologic examination, visual analogue scale (VAS) above 4 points (out of 10), willingness to participate in the study, and not receiving concurrent medical treatment.

Exclusion criteria were any type of injection or physical treatment in the last 3 months, sacroiliac joint problems based on the physical examination, bertolotti syndrome, hemorrhagic disorders, systemic infection or local infection at injection site, positive history of significant allergic reactions to corticosteroids, pregnancy, diabetes, dynamic listhesis, and body mass index (BMI) 30 kg/m^2^ and more.

### Assignment of interventions

Participants were randomized by a statistician in clinic with a 1:1 ratio, using Random Allocation Software. Blocking method was used to confirm similar numbers of participants who were allocated to the two treatment arms. Random permuted block sizes of 4–6 were used. The participant allocations were kept in sealed opaque envelopes. A statistician, who was blinded to all clinical data, performed the allocation. The individuals performing the clinical tests and also the person who performed all the statistical analyses were blinded to group allocation. Neither the participants nor the person performing the intervention were blinded.

### Intervention

Patients in ESWT group, received five sessions of radial shock wave therapy (rESWT) (one session per week) with ballistic, high energy pulses through three weeks via a Zimmer enPulsPro Medizin System Gmbh, Germany. Patients’ position was prone, affected side was exposed, and the applicator was directed in the most tender point over the lower back affected side and gently moved around the trigger point in each treatment session. Transmission gel was applied between the device and the subjects’ skin with no local anesthetic. rESWT was used with shockwaves of 1500 pulses/session with an energy flux density of 0.1 mj/mm^2^_/_min, energy level of 2–4, a frequency of 10–16 Hz, and pulse rate of 160/min in total, based on the recommended treatment protocol of enPulsPro System for myofascial syndrome.

The participant in corticosteroid TPI group was positioned in a prone situation and trigger points on the QL were checked. Injections on trigger points were done based on the method explained by Travell and Simons [20, 21]. Antisepsis was applied and trigger point was confirmed to be held immobilize between two fingers. Then, a sterile 5 ml syringe containing 40 mg triamcinolone + 2 ml of lidocaine 2% was inserted with an angle of 90° into the skin through the skin and progressed onward to a depth of 3–3.5 cm till the trigger point was grasped. The trigger point was recognized by local twitch reaction or tightening of the taut band with pain. Then, the needle was retreated to the subcutaneous tissue and forwarded to different directions (superiorly, inferiorly, laterally, and medially) with fan-shaped syringe turning after some material was thrown in after negative aspiration [22, 23]. At most, two trigger points were injected in each participant. To promote hemostasis, the inserted locations were compressed by hand for two minutes. Subsequently, the participant was altered to a supine situation and remained for duration of ten minutes.

Stretching exercises were instructed for participants in both groups and activities of daily living modifications (e.g. avoiding heavy lifting, walking long distances, and high-impact exercises) were also taught. Patients were advised to use only acetaminophen for pain relief in the event of severe pain and the number of pills used was documented. A diary was applied to note the stretching exercises dates and durations (minute).

### Outcome measures

Study variables included VAS, pressure-pain threshold (PPT), Oswestry disability index (ODI), and the short form (36) health survey (SF-36), which were measured before the intervention, two weeks after, and four weeks after the intervention. The study outcome was defined as a significant reduction in pain intensity based on VAS, or a significant improvement in Lumbar functional status based on ODI, or an improvement in PPT based on a digital algometer measurement or an increasing in quality of life based on SF-36 score.

### Visual analogue scale (VAS)

Pain intensities in both groups were assessed using VAS, which is a widely used test in pain assessment studies with acceptable reliability and validity (0–10 cm pain scale: 0 = none and 10 = unbearable) [24]. Accordingly, a minimal improvement of 30% in VAS was defined as Minimal Clinically Important Difference (MCID) [25].

### Pressure-pain threshold (PPT)

**Fig. 1:**
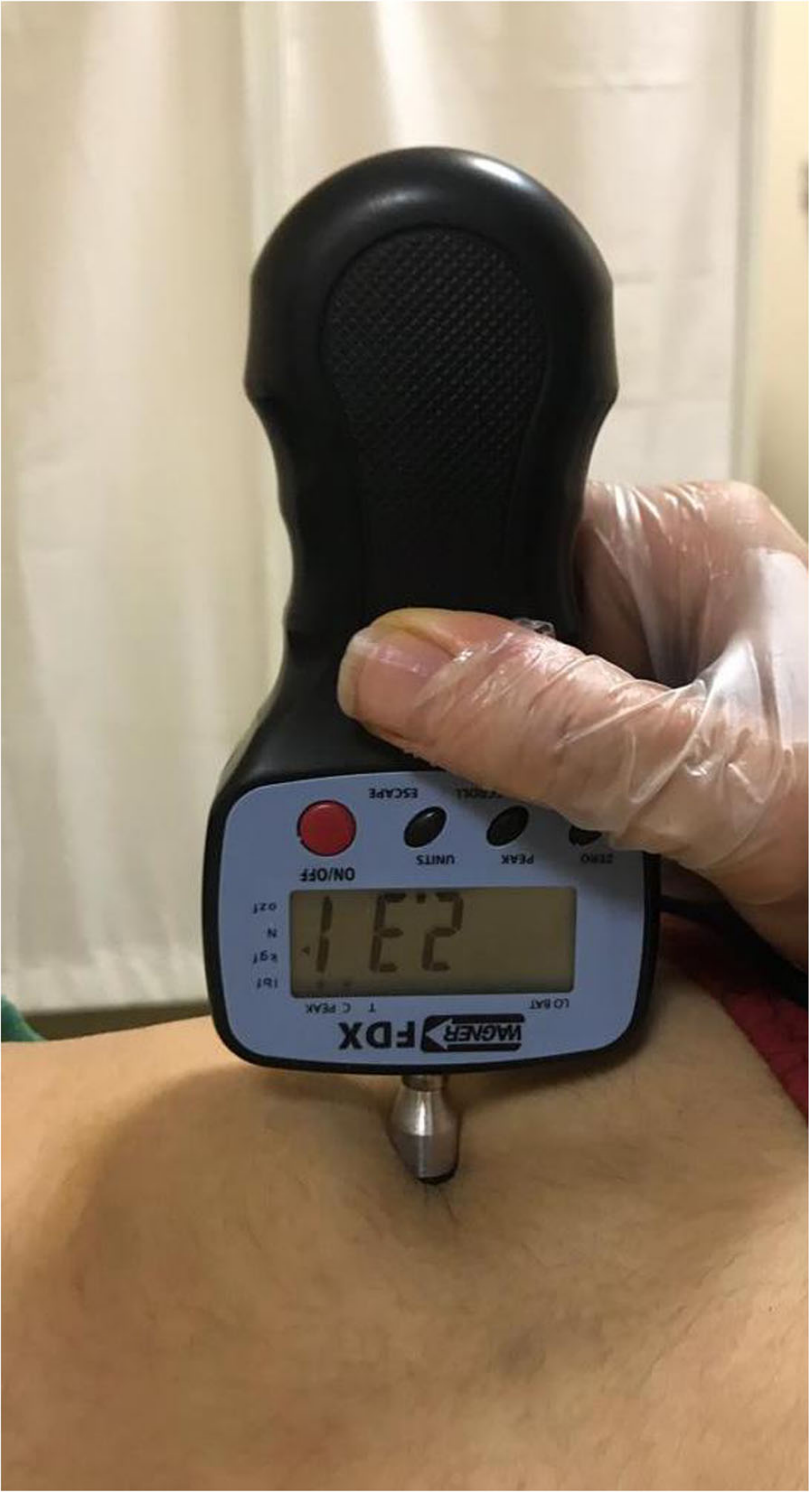
Algometry measurement on inferior trigger points in the quadratus lumborum muscle

A digital algometer (Wagner Instruments, Greenwich, CT, USA) enabled us to assess the PPT in trigger points of the QL muscle [26]. Larger values indicate higher pain thresholds. The algometer circular flat tip with 1.0 cm^2^ surface was slowly pushed upright to the skin over the trigger points until the subject’s expressed sensation altered from compression to the pain. The exerted pressure was enlarged at a rate of 1 kg/cm2. Participants were requested to inform the assessor by saying “yes” when the pain was perceived. The measurements were implemented three times with 40 s intervals, and the mean average value was considered (Fig. 1) [27]. A mean difference of 0.94 kg/cm^2^ in PPT was defined as MCID [28].

### Oswestry disability index (ODI)

Lumbar functional status was assessed using ODI questionnaire, which is a routinely used index for LBP, and the reliability and validity of its Persian version have been established. ODI ranges from 0 to 100, with lower scores indicating less severe symptoms [29]. This questionnaire consists of 10 items, and each item is scored from 0 to 5. To calculate the disability parentage, the score was divided to 50 and multiplied by 100. The score ranges from a minimum of zero, which indicates no disability, up to a maximum of 50, which indicates 100% disability. The items involve “pain intensity, personal care, lifting, walking, sitting, standing, sleeping, sex life, social life, and traveling”. A minimal improvement of 30% in ODI was considered as MCID in the present study [28].

### Quality of life (QoL)

Quality of life status was evaluated using the short form (36) health survey (SF-36) questionnaire, which consists of 36 items regarding the quality of life with respect to the physical and emotional aspects. Likert scales and yes/no options were used to assess function and well-being on this 36-item questionnaire. To score the SF-36, scales are standardized with a scoring algorithm to obtain a score ranging from 0 to 100. Higher scores indicate better health status. Also, the scoring method was mentioned in the form. This questionnaire has been translated to Persian and the reliability and validity of the Persian version of the questionnaire have been assessed [30]. A minimal improvement of 20% in SF-36 scores were defined as MCID in the present study [31].

### Side effects and adverse events

At the second and fourth weeks of follow-up, participants completed a form on the side effects they had endured with study interventions. The form asked about frequently expressed side effects (such as discomfort).

### Sample size

The study sample size was determined regarding to the main outcome of the study: “improvement in the VAS”. Considering the VAS, a group with a sample size of 21 was necessary to achieve a between group mean difference of 1.43 points [32], on a significance level (alpha) of 0.05 and power of 97.5%. Considering 25% loss to follow-up rate, a sample size of 27 patients in each treatment group was determined for this trial [33, 34].

### Statistical analysis

All information obtained from the study were screened and analyzed by the SPSS 20.0 software (IBM SPSS Statistics for Windows, Version 20.0. Armonk, NY: IBM Corp). Normality assumption analysis was performed using of Kolmogorov Smirnov test. In bivariate analysis, independent samples t-test was used for numerical scales, and χ^2^ test or fisher’s exact test were used for categorical scales. To evaluate within group changes and between group differences, two-way mixed ANOVA test along with the sidak posthoc as adjustment procedure were used. Intention-to-treat (ITT) principle in which study participants are analyzed according to their randomized assignment even if they were lost to follow up or failed to adhere to the protocol was applied in this study.

We reported effect size in terms of Cohen’s d for all outcome measures. We used on benchmarks suggested by Cohen to interpret the calculated effect sizes as small (d = 0.2), medium (d = 0.5), and large (d = 0.8) [35]. The Number Needed to Treat (NNT) is a measure of treatment effect, which represent the number of patients need to be treated to prevent one additional bad outcome. RR is used to compare the risk of an outcome when receiving a medical treatment versus no treatment (or placebo). Risk ratio (RR) and NNT along with 95% CI were calculated. To produce the 95% CI, the exact method of estimation was used. In this trial, the general strategy for analysis was based on an ITT approach. A *P* value less than 0.05 was considered as statistically significant. Also, the graphs were provided by GraphPad prism 6.0.

## Results

### Study population

**Fig. 2:**
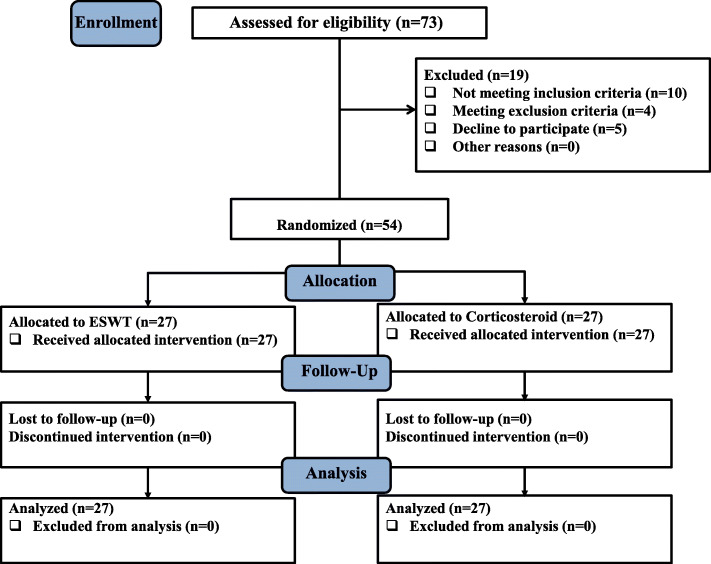
Flowchart

A total of 73 patients who were referred with the dominant complaints of LBP from January to December 2019 were included in this study. Out of these 73 patients, 19 were excluded, including 10 failures to confirm the inclusion criteria, 4 conforming the exclusion criteria, and 5 declined to participate in the study. Eventually, 54 patients were enrolled in the study. The recruited patients were randomly assigned to ESWT and TPI groups, in a ratio of 1:1. No patient was excluded from the analysis; finally, there were 27 patients in each group. The study flowchart is presented in Fig. 2. Additional basic and demographic characteristics of the participants are shown in Table 1. None of the participants used acetaminophen or any other pain killers through the study period.

**Table 1:** Demographic and baseline characteristics comparison of patients with chronic LBP with trigger point on quadratus lumborum muscle between ESWT or corticosteroid TPI groups (*n* = 54)

Characteristics	ESWT (n = 27)	Corticosteroid TPI (*n* = 27)	*P* value
Gender (female)	20 (74.1%)	17 (63.0%)	0.379^a^
Age, yrs	44.74 ± 9.34	45.04 ± 11.86	0.919^b^
BMI, kg/m^2^	27.47 ± 1.44	26.20 ± 2.06	0.110^b^

Note: The data are presented as mean ± standard deviation or frequency (percentage)

*Abbreviation*: *ESWT* Extracorporeal shockwave, *TPI* Trigger point injection, *BMI* Body mass index

Symbol: ^a^ P obtained from Chi-Square test, ^b^P obtained from Independent samples t-test

### Effects of the intervention

There was a significant interaction between time points (0, 2 week, 4 week) serving as the within-group factor and group (ESWT vs. corticosteroid TPI) as the between-group factor with regard to study variables (ODI: F (time*group) _(2, 104)_ = 26.42, *P* <  0.001; PPT: F (time*group) _(2, 104)_ = 14.87, P <  0.001; SF-36: F (time*group) _(2, 104)_ = 9.96, P <  0.001; VAS: F (time*group) _(2, 104)_ = 30.90, P <  0.001). As there is a statistically significant interaction, we needed to determine the difference between study groups at each level of time factor. The results on the clinical outcomes are shown in Tables 2 and 3.

**Table 2:** Mean Changes from the baseline in the clinical values of patients with chronic LBP with trigger point on quadratus lumborum muscle within the each group of ESWT or corticosteroid TPI (n = 54)

Clinical value		ESWT(n = 27)		Corticosteroid TPI (n = 27)	
		Mean ± SD	MC (95% CI) *P* value †	Mean ± SD	MC (95% CI) P value †
ODI	Before	22.44 ± 1.44	Reference	23.93 ± 1.27	Reference
	Week 2	16.07 ± 1.29	6.37 (4.34 to 8.40) < 0.001	13.52 ± 1.13	10.41 (8.38 to 12.43) < 0.001
	Week 4	14.26 ± 1.32	8.18 (6.02 to 10.35) < 0.001	19.89 ± 1.13	4.04 (1.87 to 6.20) 0.001
PPT	Before	28.04 ± 0.75	Reference	29.41 ± 0.47	Reference
	Week 2	33.37 ± 1.21	5.33 (3.43 to 7.23) < 0.001	38.70 ± 0.83	9.30 (7.40 to 11.20) < 0.001
	Week 4	34.26 ± 1.23	6.22 (4.26 to 8.19) < 0.001	34.37 ± 0.70	4.96 (3.00 to 6.93) < 0.001
SF-36	Before	74.59 ± 1.35	Reference	73.78 ± 1.30	Reference
	Week 2	81.93 ± 1.53	7.33 (4.18 to 10.48) < 0.001	79.56 ± 1.46	5.78 (2.63 to 8.93) < 0.001
	Week 4	84.00 ± 1.48	9.41 (5.93 to 12.88) < 0.001	75.48 ± 1.08	1.70 (− 1.76 to 5.18) 0.546
VAS	Before	7.63 ± 0.27	Reference	7.22 ± 0.26	Reference
	Week 2	5.81 ± 0.25	1.81 (1.18 o 2.44) < 0.001	3.52 ± 0.35	3.70 (3.07 to 4.33) < 0.001
	Week 4	5.11 ± 0.36	2.52 (1.90 to 3.13) < 0.001	5.44 ± 0.27	1.78 (1.16 to 2.39) < 0.001

*Abbreviations*: *ESWT* Extracorporeal shockwave, *TPI* Trigger point injection, *SD* standard deviation, *MC* mean change, *CI* confidence interval, *ODI* Oswestry disability index, *PPT* Pressure Pain Threshold, *SF-36* Short Form (36) Health Survey, *VAS* Visual Analogue Scale

Symbols: †*P* values obtained from Mixed ANOVA test

**Table 3:** Number of patients achieved study outcomes^a^ and comparison of relative risk (RR) and number needed to treat (NNT) between the two groups based on study outcomes (n = 54)

Clinical Values		ESWT (n = 27)	Corticosteroid TPI (n = 27)	MD _(ESWT-Corticosteroid)_(95% CI) *P* Value †
		Mean ± SD	Mean ± SD	
ODI	Week 2	6.37 ± 0.75	10.41 ± 0.89	−4.56 (−6.01 to − 0.89) 0.007
	Week 4	8.18 ± 1.05	4.04 ± 0.66	4.63 (1.14 to 8.11) 0.002
PPT	Week 2	5.33 ± 0.76	9.30 ± 0.78	−4.33 (− 7.27 to − 3.40) 0.001
	Week 4	6.22 ± 0.89	4.96 ± 0.69	1.11 (− 1.94 to 3.72) 0.379
SF-36	Week 2	7.33 ± 1.35	5.78 ± 1.20	2.37 (− 6.62 to 1.87) 0.672
	Week 4	9.41 ± 1.71	1.70 ± 1.02	8.12 (4.83 to 12.20) < 0.001
VAS	Week 2	1.81 ± 0.16	3.70 ± 0.32	−2.30 (−3.16 to − 1.43) < 0.001
	Week 4	2.52 ± 0.29	1.78 ± 0.19	0.71 (−0.36 to 1.23) 0.109

*Abbreviations*: *ESWT* Extracorporeal shockwave, *TPI* Trigger point injection, *SD* standard deviation, *MD* mean difference, *CI* confidence interval, *ODI* Oswestry disability index, *PPT* Pressure Pain Threshold, *SF-36* Short Form (36) Health Survey, *VAS* Visual Analogue Scale

Symbols: †*P* values obtained from Mixed ANOVA test

### Within group comparison of clinical outcomes

The within group comparisons revealed that, at two weeks follow-up time-point, both groups experienced statistically significant clinical benefits in their ODI (ESWT: two-week MC (95% CI); *P*-value: 6.37 (4.34 to 8.40); < 0.001, four-week MC (95% CI); *P*-value: 8.18 (6.02 to 10.35); < 0.001, Corticosteroid TPI: two-week MC (95% CI); *P*-value: 10.41 (8.38 to 12.43); < 0.001, four-week MC (95% CI); *P*-value: 4.04 (1.87 to 6.20); < 0.001), PPT (ESWT: two-week MC (95% CI); *P*-value: 5.33 (3.43 to 7.23); < 0.001, four-week MC (95% CI); P-value: 6.22 (4.26 to 8.19); < 0.001, Corticosteroid TPI: two-week MC (95% CI); P-value: 9.30 (7.40 to 11.20); < 0.001, four-week MC (95% CI); P-value: 4.96 (3.00 to 6.93); < 0.001), SF-36 (ESWT: two-week MC (95% CI); P-value: 7.33 (4.18 to 10.48); < 0.001, four-week MC (95% CI); P-value: 9.41 (5.93 to 12.88); < 0.001, Corticosteroid TPI: two-week MC (95% CI); P-value: 5.78 (2.63 to 8.93); < 0.001, four-week MC (95% CI); P-value: 1.70 (− 1.76 to 5.18); < 0.001), and VAS scores (ESWT: two-week MC (95% CI); P-value: 1.81 (1.18 to 2.44); < 0.001, four-week MC (95% CI); P-value: 2.52 (1.90 to 3.13); < 0.001, Corticosteroid TPI: two-week MC (95% CI); P-value: 3.70 (3.07 to 4.33); < 0.001, four-week MC (95% CI); P-value: 1.78 (1.16 to 2.39); < 0.001) compared to the before treatment, which lasted for 4 weeks after the treatment. The only considerable point was in the quality of life of the patients under corticosteroid injection. Although corticosteroid injections could improve the patient’s quality of life within two weeks after the treatment (*P* value< 0.001), this change did not last for one month (*P* value = 0.546). The ESWT group reached a peak in therapeutic effects four weeks after the treatment, and the changes were statistically significant compared to before treatment. Reaching a peak in therapeutic effects for corticosteroid injection group occurred two weeks after the treatment, and the changes were statistically significant compared to before treatment (Table 2).

### Between group comparison of clinical outcomes

The between group comparison at two weeks after treatment indicated that, corticosteroid TPI group recorded statistically significant more improvements of ODI, VAS, and PPT scores compared to the ESWT group (All P value< 0.05). Compared to the TPI group, participants in the ESWT group experienced significantly more improvements in the ODI and SF-36 at 4th week post treatment evaluations (P value< 0.05) (Table 3).

### Study outcomes evaluation

Based on the commonly used interpretation of the Cohen’s d values, the results refer to large effect size for all study outcomes in ESWT group (d = 4.72, d = 1.58, d = 5.48, and d = 7.47 for ODI, PPT, SF-36, and VAS, respectively).

The results showed that 59.31% of patients in the ESWT group achieved the primary outcome (achieving > 30% reduction from base line at 4th week follow up) versus 25.9% in the corticosteroid TPI group: 16 (59.31%) vs. 7 (25.9%), respectively; RR = 1.46 with 95% CI = 1.04 to 2.19; NNT = 3 with 95% CI = 1.85 to 14.4; *P* = 0.013). NNT = 3 indicates that we would, on average, have to teat 3 patients for 1 patient to have been experienced 30% or more pain relief at 4th week follow up (Table 4).

**Table 4:** Comparison of Relative Risk (RR) and Number needed to treat (NNT) between the two groups based on study outcomes in participants (n = 54)

Outcome ^a^	ESWT (n = 27)	Corticosteroid TPI (n = 27)	P value†	RR (95% CI)	NNT (95% CI)
ODI	17 (63.0%)	4 (14.8%)	< 0.001	2.67 (1.53 to 4.66)	2 (1.35 to 3.61)
PPT	23 (85.20%)	18 (66.70%)	0.111	1.82 (0.84 to 2.18)	5.4 (2.54 to 22.4)
SF-36	10 (37.0%)	1 (3.7%)	0.002	2.30 (1.62 to 43.26)	2 (1.35 to 3.45)
VAS	16 (59.3%)	7 (25.9%)	0.013	1.46 (1.04 to 2.19)	3 (1.85 to 14.4)

Note: The data are presented as frequency (percentage)

*Abbreviations*: *ESWT* Extracorporeal shockwave, *TPI* Trigger point injection, *RR* Relative risk, *NNT* Number Needed to Treat, *CI* confidence interval (calculated using exact method), *ODI* Oswestry disability index, *PPT* Pressure Pain Threshold, *SF-36* Short Form (36) Health Survey; VAS: Visual Analogue Scale

Symbols: ^a^The outcomes is defined as at least 30% reduction in the final score (after 4 weeks) of VAS compared with the baseline score, OR at least 30% reduction in the final score (after 4 weeks) of ODI compared with the baseline score, OR a mean difference of 0.94 kg/cm^2^ (after 4 weeks) of PPT compared with the baseline score, OR at least 20% increase in the final score (after 4 weeks) of SF-36 compared with the baseline score; †obtained from χ^2^ Chi-Square test or Fisher’s exact test

The number of the patients achieved MCID concerning ODI was significantly higher in ESWT group. The patients in the ESWT group were 2.67 times more likely to achieve 30% reduction in ODI compared to the corticosteroid TPI group: 17 (63.0%) vs. 4 (14.8%); RR = 2.67 with 95% CI = 1.53 to 4.66; NNT = 2 with 95% CI = 1.35 to 3.61 (*P* < 0.001). For additional evaluation of the effectiveness of ESWT, we calculated the NNT to achieve 30% or more reduction in ODI. An NNT = 2 for ODI indicates that if two patients are treated with the ESWT, one would achieve MCID concerning ODI at 4th week follow up and was statistically significant compared with corticosteroid TPI (Table 4).

The number of the patients achieved MCID regarding PPT (a mean difference of 0.94 kg/cm2) in the ESWT group was more than the corticosteroid TPI group, however, the result was not statistically significant between the study groups (23 (85.20%) vs. 18 (66.70%); RR = 1.82 with 95% CI = 0.84 to 2.18; NNT = 5.4 with 95% CI = 2.54 to 22.4; *P* = 0.111) (Table 4).

The between group differences were statistically significant in favor of the other outcome, achieving 20% improvement (MCID) in SF-36 (10 (37.0%) vs. 1 (3.70%); RR = 2.30 with 95% CI = 1.62 to 43.26; NNT = 3 with 95% CI = 1.85 to 14.4; *P* < 0.01). For every three patients, ESWT cause one patient to achieve 20% or more improvement in SF-36 (Table 4).

### Side effects and adverse events

No clinically important adverse events, side effects, or severe complications (e.g., hematomas and other abnormal musculoskeletal events), which required medical interference, were stated in either groups.

## Discussion

In present study, we compared the efficacy of rESWT (shockwaves of 1500 pulses/session with an energy flux density of 0.1 mj/mm^2^_/_min and a frequency of 10–16 Hz) and corticosteroid TPI (40 mg triamcinolone) in alleviating pain and improving disability, PPT and quality of life in the patients with MPS in the QL muscle, and found that corticosteroid TPI resulted in significantly higher improvements of disability, pain intensity and PPT compared to the ESWT at two weeks follow-up time-point. However, after 4 weeks, participants in the ESWT group experienced further improvements regarding the disability and quality of life compared to the corticosteroid TPI group. Accordingly, this provides the evidence of the short-term beneficial effects of corticosteroid injection on pain intensity, PPT and disability. However ESWT was more effective in management of disability and improving the quality of life at one-month follow-up after treatment.

LBP is the second most prevalent condition that affects adult populace [36]. If this disorder is left untreated, it may significantly disturb quality of life in patients [37]. In general, management approaches of LBP comprise pharmacological and non-pharmacological treatments [38]. Pharmacological treatments have inadequate efficacy and a wide range of adverse events [39]. Therefore, present guidelines principally emphasis on the non-pharmacological approaches [40].

ESWT has been considered as a noninvasive non-pharmacological treatment of many musculoskeletal disorders in recent decades, in spite of its uncertain mechanism of action [41–43]. The utilization of rESWT in MPS has not been completely investigated. Nonetheless, there is several evidence concerning the beneficial effect of rESWT for plantar fasciitis, calcific tendinitis and epicondylitis [44, 45]. The review of the existing literature reveals that, in spite of the novelty and high acceptance, there are only a few of randomized clinical trials (RCTs) on the effectiveness and safety of ESWT in LBP patients [32, 46–48].

In a similar study to our trial, Hong et al. [14] compared the efficiency of ESWT for a total of 3 times, at 3-day intervals and TPI in 30 patients with MPS in the QL. Contrary to the findings of the present study, ESWT was found to be more effective than TPI for pain relief. However, ESWT was not better than TPI with respect to disability. While, in our study, TPI was found to be more effective than ESWT in improving disability at 2nd week assessment and ESWT was found to be more effective than corticosteroid TPI in improving disability at 4th week assessment. After 4 weeks of the treatment, higher percent of the participants in ESWT group achieved MCID concerning disability index. ESWT was related with at least two time higher likelihood of such an improvement in treated patients compared to corticosteroid TPI. Two patients must be treated with the purpose of expect that one patient will experience ≥30% improvement concerning disability at 4th week assessments that was statistically significant compared to corticosteroid TPI.

Lee et al. [49] in comparison the effectiveness of ESWT and TPI in 31 patients with MPS in the trapezius muscle concerning decreasing pain intensity and improving of PPT, found no significant differences between ESWT and TPI in terms of VAS and PPT. In our study, TPI was more effective than ESWT in improving pain intensity and PPT at 2 week evaluations. These different findings may be due to applying different intensities and impulses of ESWT. However after 4 weeks, their efficiency was comparable. A considerably greater percentage of participants treated with ESWT achieved MCID concerning VAS score after 4 weeks of the treatment. In other words, after 4 weeks treatment, participants in ESWT group were more likely to have such an improvement compared to corticosteroid TPI. Three patients must be treated to anticipate that one patient would experience ≥30% decrease in pain intensity at 4th week assessments that was statistically significant compared to corticosteroid TPI.

In other studies, ESWT has been established as an efficient treatment approach in decreasing resting and movement pain and disability, and also improving the quality of life and depression compared to placebo [50] and conservative physical therapies [47] in patients with chronic LBP. In our study, significantly higher proportion of participants treated with ESWT compared with corticosteroid TPI achieved MCID concerning quality of life at week 4 of study. After 4 weeks treatment, participants in ESWT group were at least two times more likely to have such an improvement in SF-36 score. Two patients must be treated with the purpose of expect that one patient will experience ≥20% improvement regarding quality of life at 4th week assessments that was statistically significant compared to corticosteroid TPI.

In the prospective, randomized study by Walewicz et al. [51] on a total of 52 patients with LBP, rESWT (2000 pulses; 2.5 bars; 5 Hz, 7 mins) twice a week for five weeks (10 sessions) was predominantly effective in the long-term management and provides a stable advantageous effect for patients without unexpected relapse. While, in short-term the pain relief was not considerable immediately after rESWT.

Several studies have attempted to clarify the mechanism of shock waves from the basic science and clinical studies. The thorough mechanisms for its pain-relieving and functional properties are not completely figured out. MPS is a condition in which stimulation of trigger points in the muscles can lead to referred discomfort and pain. So, it is critical to remove the trigger points. The ESWT, acting through electromagnetic stimulation mechanism, generates waves with low energy level that can be impressive by enhancing the blood circulation in the treated location [13, 52]. The transferred waves lead to tissue repair through making micro-traumas and releasing of growth and molecular factors [53]. Additionally, ESWT stimulates the A delta receptor, which provides prompt neuro-stimulator conduction and this represses C fiber. The C fiber induction slow neuro-stimulator conduction; and therefore, its repression blocks the nerve transmission [54]. Furthermore, it has been suggested that ESWT can decrease the pain in the tissues via selective demolition of the non-myelinated fibers and is fruitful in decreasing the concentration of substance P, in addition to decreasing the production of substance P in the dorsal root ganglia [55, 56]. ESWT can also decline the concentrations of the inflammatory cytokines (interleukins (IL) and matrix metalloproteinase (MMP)) [57].

In spite of its relatively high cost (equipment and space requirements), ESWT may be endorsed as a treatment alternate for myofascial trigger points. ESWT has numerous excellences to other approaches as follows: It is non-aggressive, infection free, and easy for using in outpatient backgrounds. Furthermore, it is probable to adjust treatment protocol based on the acceptance and compliance of patients. ESWT can be applied to larger surface by moving the probe location. In general, the safety of ESWT is evidently confirmed through the cumulative data [58]. The important thing to be considered is that, TPI is not always reliable of exhaustively addressing diffused several taut bands. In such cases, ESWT is an applicable treatment approach, with wide exposure and no post-injection discomfort.

This study has some recommendations for the use of rESWT in management of patients with LBP because of the MPS of QL. When rESWT is applied to manage the complaints of patients with LBP because of MPS, it provides more beneficial effect concerning improving disability and quality of life in the medium term with a stable effect compared to corticosteroid TPI. However, in short-term the results are best for corticosteroid TPI regarding pain reduction and improving disability and PPT. That means rESWT does not result in significant pain relief effects immediately after the treatment. We recommend rESWT with 1500 pulses/session; energy level of 2–4; 10–16 Hz, 7 mins; performed one session per week for five weeks for patient with MPS of QL.

Limitations of this study were firstly the absence of a control group to rule out the placebo effect, or the lack of a group to receive both interventions. Secondly, because this study was performed for a short period of time, it was not capable to entirely evaluate the long-term effects of each intervention. Therefore, future trials should be designed for a longer period of time to completely assess the effects.

## Conclusion

In MPS patient with inferior trigger points of QL muscle, corticosteroid TPI was more efficient than ESWT in decreasing pain intensity and disability after 2 week treatment. However, after 4 week treatment, ESWT was more efficacious than corticosteroid TPI in improving quality of life and disability and was related with more likelihood of at least 30% decrease in pain intensity and disability and at least 20% improvement in quality of life in treated patients compared to corticosteroid TPI. The number of patients who must be treated by ESWT in order to expect that one patient will achieve such improvements in pain intensity; disability and quality of life were 3, 2 and 2 over a 4 week treatment period that were statistically significant compared to corticosteroid TPI.

## Data Availability

All the necessary data are presented herewith. However if needed, raw data on excel format can be availed on reasonable request from the corresponding author.

## Abbreviations

BMI: Body mass index
CI: Confidence interval
ESWT: Extracorporeal shockwave
LBP: Low back pain
ODI: Oswestry disability index
MD: Mean difference
PPT: Pressure Pain Threshold
QL: Quadratus lumborum
SD: Standard deviation
SF-36: Short Form (36) Health Survey
VAS: Visual Analogue Scale
